# Temporal Profile of Soluble TLR4 and its Association with Intracerebral Haemorrhage Expansion

**DOI:** 10.1007/s12975-026-01473-2

**Published:** 2026-07-20

**Authors:** Maria Lucas-Parra, Carme Gubern-Mérida, Sofia Dávila, Tomàs Xuclà-Ferrarons, Joan Martínez, Saima Bashir, Alan Murillo, Víctor Vera-Monge, Juan Álvarez-Cienfuegos, Laia Carballo-Perich, Elisabet Ortiz, Isabel Vielba-Gomez, Joaquín Serena, Mikel Terceño, Yolanda Silva

**Affiliations:** 1 Cerebrovascular Pathology Research Group, Institut d’Investigació Biomèdica de Girona (IDIBGI-CERCA), RICORS-ICTUS, Parc Hospitalari Martí i Julià, Edifici M2, Salt, 17190 Spain; 2 Department of Biology, University of Girona (UdG), Maria Aurèlia Capmany, 40, Girona, 17003 Spain; 3 Medical student, University of Girona (UdG), Emili Grahit, 77, Girona, 17003 Spain; 4 Stroke Unit, Department of Neurology, Cerebrovascular Pathology Research Group, University Hospital Doctor Josep Trueta, Institut d’Investigació Biomèdica de Girona (IDIBGI-CERCA), RICORS-ICTUS, Av. França s/n (7a planta), Girona, 17007 Spain; 5 Statistics Unit, Institut d’Investigació Biomèdica de Girona (IDIBGI- CERCA), Parc Hospitalari Martí i Julià, Edifici M2, Salt, 17190 Spain; 6 Department of Medical Science, University of Girona (UdG), Emili Grahit, 77, Girona, 17003 Spain

**Keywords:** Soluble toll-like receptor 4, Haematoma expansion, Intracerebral haemorrhage, Biomarkers, Prognosis

## Abstract

Inflammation contributes to haematoma expansion (HE) and poor prognosis in spontaneous intracerebral haemorrhage (ICH). This study aimed to characterise the temporal profile of soluble Toll-like receptor 4 (sTLR4) during the hyperacute and acute phases of ICH and to evaluate its association with HE. Serum sTLR4 levels were measured at admission, 24 and 72 h from 99 patients with primary hemispheric ICH within 12 h of symptom onset, and at baseline from 39 non-stroke controls using enzyme-linked immunosorbent assay (ELISA). Longitudinal sTLR4 fluctuations were evaluated through linear mixed-effects models. Independent HE prognosticators were identified using multivariable logistic regression, and model performance was assessed via the area under the receiver operating characteristic curve (AUC-ROC). ICH patients exhibited significantly higher baseline sTLR4 levels than controls, which declined over time and were not associated with clinical variables. Notably, patients with HE demonstrated higher admission sTLR4 levels than those without HE (2.50 [95% CI: 1.84–3.16] vs. 1.62 [95% CI: 1.22–2.02] ng/mL, *p* = 0.027). Furthermore, while the non-HE group showed a rapid decrease within the first 24 h, the HE group maintained elevated levels with a more gradual decline. A multivariable model incorporating baseline ICH volume and sTLR4 levels achieved an AUC of 0.829, with positive and negative predictive values of 70.8% and 90.1%, respectively. These findings suggest that TLR4 may be involved in the pathophysiology of HE, and the combination of baseline ICH volume and sTLR4 levels could serve as a useful tool to stratify patients into low- and high-risk groups for HE.

**Supplementary Information:**

The online version contains supplementary material available at https://doi.org/10.1007/s12975-026-01473-2.

## Introduction

Spontaneous intracerebral haemorrhage (ICH) remains among the most devastating subtypes of stroke, accounting for a disproportionate amount of stroke-related mortality and long-term disability worldwide. While it constitutes approximately 10% to 30% of all stroke cases, its acute nature and the limited efficacy of existing interventions contribute to a one-month mortality rate of nearly 40% [1].

Haematoma expansion (HE) is recognised as a vital, independent predictor of poor prognosis and the most significant modifiable factor in the hyperacute phase of the disease. The incidence of expansion ranges widely from 13% to 38%, depending on the definitions employed and the timing of follow-up imaging. HE typically occurs within the first few hours after symptom onset, representing a narrow but crucial therapeutic window [2]. Although different mechanisms have been suggested, the precise molecular pathways involved in HE are still poorly understood [3–5]. A more comprehensive understanding of its pathophysiology is therefore essential. Moreover, given the impact of HE on patient prognosis, considerable efforts have been devoted in recent years to the identification of reliable predictors of this phenomenon.

Among the imaging biomarkers evaluated to date, the spot sign (SS) on computed tomography angiography (CTA) has shown the greatest predictive potential for HE [6–9]. However, its use as an inclusion criterion in anti-HE clinical trials has not yielded positive results until now [10]. With regard to molecular biomarkers, previous studies have demon-strated that inflammatory markers in the early acute phase of ICH are associated with subsequent enlargement of the haematoma and poor outcome [6, 11–13]. Traditionally, HE has been considered a mechanical event defined by vascular rupture and subsequent mass effect. Nevertheless, contemporary understanding of ICH has evolved toward a model where neuroinflammation is not merely a response to damage, but a key driver of HE and secondary brain injury. The immediate release of blood-derived components, including thrombin and iron, acts as a danger signal that activates pattern recognition receptors in microglia and astrocytes [14]. The resulting acute inflammation promotes the release of mediators that further compromise the structural integrity of neighbouring small vessels, thereby facilitating a cascade of microvascular rebleeding and progressive HE [15].

Toll-like receptor 4 (TLR4) plays a central role in orchestrating this immune response by recognising signals of tissue injury and translating them into a coordinated inflammatory cascade [16, 17]. TLR4 is expressed on the cell membranes of microglia, astrocytes, neurons, and cerebral endothelial cells and triggers the synthesis of pro-inflammatory cytokines and chemokines [17, 18]. Although it has been widely studied in ischaemic stroke, the role of TLR4 in haemorrhagic stroke has received comparatively limited attention, with most available evidence derived from preclinical models [19–22]. In experimental ICH, pharmacological inhibition or genetic deletion of TLR4 has been shown to promote haematoma resolution, reduce inflammatory cytokine release, and improve neurological outcomes. In clinical studies, higher baseline TLR4 expression in circulating monocytes has been independently associated with worse functional outcome at three months after ICH [23]. Nevertheless, the specific contribution of TLR4 to HE remains insufficiently explored.

More recently, soluble TLR4 (sTLR4), has emerged as a potential biomarker of interest due to its detectability in body fluids [17]. To date, only a single study has assessed circulating sTLR4 levels in an Asian cohort of ICH patients, reporting that higher plasma concentrations within 48 h of symptom onset were associated with larger haematoma volumes and poor functional outcomes [24]. Importantly, no data are currently available regarding sTLR4 levels during the very early time window in which HE most frequently occurs.

Accordingly, the present study aimed to characterise the temporal profile of sTLR4 during the hyperacute and acute phases of spontaneous ICH and to evaluate its potential association with HE.

## Materials and Methods

This study was conducted in accordance with the declaration of Helsinki and was approved by the Ethics Committee of the Dr. Josep Trueta University Hospital (Code sTLR4-HIC). All participants signed a written informed consent form.

### Study Design and Population

We conducted a retrospective single-centre observational study including samples and data from (1) patients consecutively admitted to the Stroke Unit at the Dr. Josep Trueta University Hospital (Girona, Spain) between May 2017 and May 2022 with a diagnosis of primary spontaneous ICH who were prospectively included in the ICT collection of the Girona Biomedical Research Institute Dr. Josep Trueta (IDIBGI) Biobank, and (2) non-stroke controls, with no prior history of ischaemic or haemorrhagic stroke, matched for age and history of arterial hypertension, provided by the IDIBGI Horizontal Aging Program and the IDIBGI Biobank from the Aging Imagenomics Study, an observational study including participants living in the province of Girona (Northeast Catalonia, Spain) [25]. The inclusion criteria for ICH patients were as follows: (a) age ≥ 18 years; (b) diagnosis of acute spontaneous or anticoagulant-associated supratentorial ICH within 12 h of symptom onset; (c) pre-stroke modified Rankin Scale (mRS) < 3; (d) availability of a cranial non-contrast computed tomography (NCCT) scans at admission and at 24 h to assess HE; and (e) availability of serum samples obtained at admission (baseline) and at 24–72 h. Both controls and ICH patients with acute infectious diseases, active autoimmune diseases, chronic inflammatory diseases or ongoing immunosuppressive treatment at the time of admission or sample collection were excluded.

Relevant baseline demographics and clinical data from ICH patients (sex; age; vascular risk factors (history of hypertension, diabetes mellitus, dyslipidaemia, smoking and alcohol consumption); previous treatment with antiplatelet and anticoagulant drugs; and systolic and diastolic blood pressure) were recorded during hospital admission. Additionally, fibrinogen levels, leukocyte count, and the Systemic Immune-Inflammation Index (SII), calculated as (neutrophil count × platelet count) / lymphocyte count, were collected at admission as markers of systemic inflammation. Data from control subjects included sex, age, prior medical history of arterial hypertension, diabetes mellitus, dyslipidaemia and smoking.

### Neuroimaging and Clinical Data

All ICH patients received standard clinical care based on current international guidelines for ICH acute management. A NCCT scan was performed at admission and repeated 24 ± 3 and 72 ± 3 h after onset. A CTA was also performed at admission to rule out secondary causes of ICH. All examinations were performed on a 128-slice computed tomography (CT) scanner (Ingenuity; Philips Healthcare, Best, the Netherlands). We used the following parameters: (a) for NCCT: Helical scanning mode, 120kVp, 370mAs, 3-mm slice thickness, 1.5-mm gap, and 512 × 512 matrix; (b) for CTA (from aortic arch to cranial vertex): Helical scanning mode, 120kVp, 250mAs, 0.9-mm slice thickness, 0.45-mm gap, and 512 × 512 matrix. We acquired CTA images after an 80-mL bolus of Ultravist (300 mg l/mL) injected at 5mL/second followed by 20 mL of saline solution. Images were acquired by an experienced technician and supervised by a neuroradiologist, both of whom were blinded to clinical data. We collected the following radiological variables: onset-to-baseline CT time, haematoma volume, haematoma location (lobar or deep), presence of intraventricular haemorrhage and HE. Haemorrhage segmentation was performed using a deep learning-based algorithm and volumes were quantified by planimetric analysis [26]. Substantial HE was defined as an increase of 33% or higher or 6 mL or more in haematoma volume between baseline and 24 h on available cranial CT images.

Neurological deficit was evaluated through the National Institutes of Health Stroke Scale (NIHSS) on admission and at 24 h by qualified neurologists. Early neurological deterioration was defined as an increase of ≥ 4 points between baseline NIHSS scoring and 24 h.

### Serum Collection and sTLR4 Measurement

Blood samples from patients with ICH (obtained at admission and at 24 and 72 h) and from controls were collected and centrifuged at 3000 x g for 5 min. The resulting serum was immediately stored at − 80 °C until use. Serum sTLR4 levels were measured in 2-fold diluted serum samples using a commercial enzyme-linked immunosorbent assay (ELISA) kit (Human TLR4 ELISA Kit; catalog no. CSB-E12954h-96T; Cusabio, Wuhan, China), according to the manufacturer’s instructions.

### Statistical Analyses

Demographic, clinical and sTLR4 data were reported as follows. Categorical variables were expressed as frequencies and percentages and compared using chi-square test or Fisher’s exact test. Normally distributed data were expressed as mean ± SD and compared using the t-test or the one-way analysis of variance (ANOVA) test. Nonparametric variables were expressed as medians, with corresponding 25th and 75th (interquartile range), and compared using the Mann–Whitney test or the Krukal-Wallis test. The assumption of normality for continuous variables was assessed with the Shapiro-Wilk test.

Associations between clinical, biochemical and radiological variables and baseline sTLR4 levels were assessed using simple linear regression analysis. Results were presented as β coefficients with corresponding 95% confidence intervals (95% CI).

Longitudinal changes in sTLR4 levels were analysed using linear mixed-effects models with sampling time, HE status, and their interaction as fixed effects and a random intercept for each patient. An autoregressive covariance structure of order 1 [AR(1)] was used to model within-subject correlations over time.

Independent prognosticators of HE were identified by binary multivariable logistic regression analysis using the backward conditional method including variables that showed *p* < 0.1 in univariable analysis and those considered clinically relevant. Univariable and multivariable analysis results were expressed as odds ratios (OR) with the corresponding 95% CI. Model multicollinearity was assessed using the variance inflation factor (VIF), with values > 5 indicating significant collinearity. The prognostic performance of the defined model for HE was evaluated using the receiver operating characteristic (ROC) curve analysis and the optimal cut-off point was established according to Youden’s threshold point [J = maximum (sensitivity + specificity – 1)]. The corresponding positive and negative predictive values (PPV and NPV) were calculated based on a prevalence of HE 25%.

All analyses were two-tailed and a p-value less than 0.05 was considered significant. Statistical analyses were performed using the Statistical Package for the Social Sciences (SPSS) version 25.0 (IBM SPSS Statistics for Windows, NY, USA) and R 3.4.3 using pROC package [27]. Figures were made with GraphPad PRISM v.8 (GraphPad Software; San Diego, CA, USA).

## Results

### sTLR4 Concentration in ICH Patients and Non-Stroke Controls

**Fig. 1:**
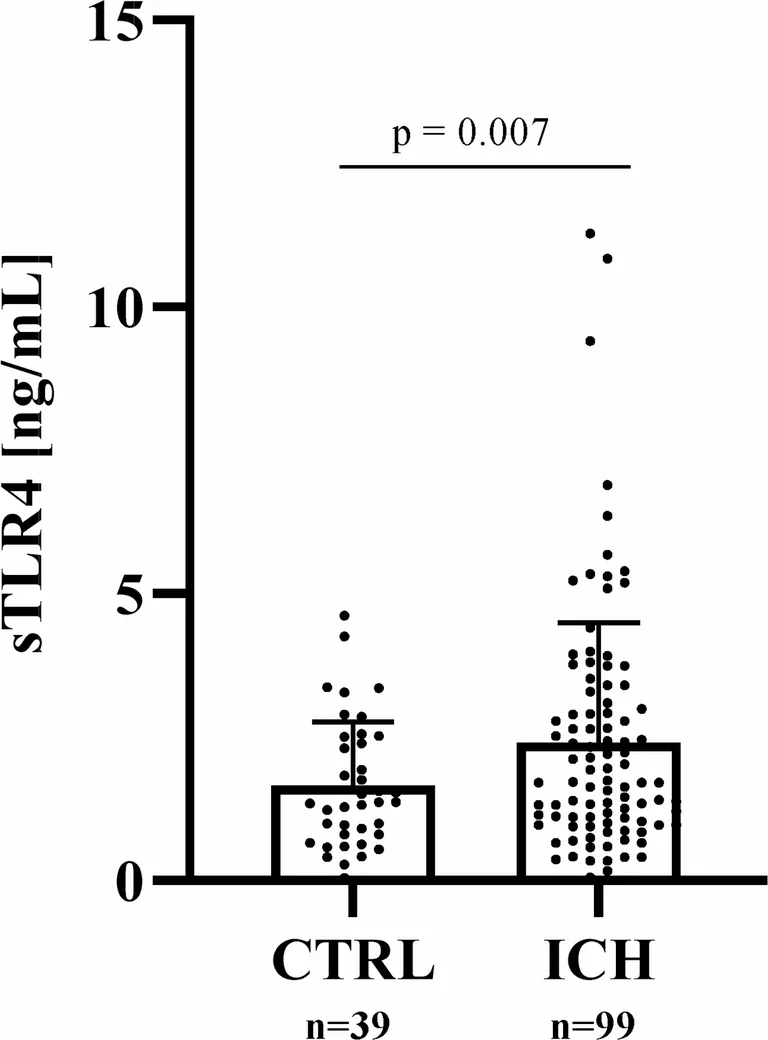
Baseline sTLR4 levels in ICH patients and non-stroke controls. Data are presented as mean ± standard deviation

From May 2017 to May 2022, 179 patients with primary ICH were enrolled in the ICT collection. Of these, 6 were excluded because the onset-to-diagnosis time exceeded 12 h, 21 due to a pre-stroke mRS > 2, 2 lacked a 24-hour NCCT scan, 37 had not serum sample at admission or it was haemolysed, and 14 had only a single serum sampling time. In total, 99 patients met all inclusion criteria (Figure S1). All 99 ICH patients and 39 non-stroke controls were included in the study. The baseline characteristics of ICH patients and controls are presented in Table 1. Compared with controls, ICH patients showed significantly higher proportions of female sex (38.4% vs. 15.4%, *p* = 0.009) and significantly lower rates of dyslipidaemia (34.3% vs. 69.2%, *p* < 0.001). Baseline sTLR4 levels were also significantly elevated in patients with ICH compared to controls (2.41 ± 2.09 ng/mL vs. 1.65 ± 1.12 ng/mL, *p* = 0.007) (Fig. 1).

**Table 1:** Baseline characteristics of patients with ICH and non-stroke controls

	ICH (*n* = 99)	Control (*n* = 39)	*p*-value
Age, years	69.7 ± 12.9	69.7 ± 5.8	0.997
Female sex	38 (38.4)	6 (15.4)	**0.009**
Hypertension	77 (77.8)	33 (84.6)	0.483
Diabetes mellitus	21 (21.2)	14 (35.9)	0.085
Dyslipidaemia	34 (34.3)	27 (69.2)	**< 0.001**
Current smoking	19 (19.8)	7 (17.9)	1.000

Categorical variables are expressed as n (%) and numerical variables as mean ± standard deviation

### sTLR4 Concentration and Clinical Characteristics of ICH Patients

The mean age of the patients with ICH was 69.7 ± 12.9 years. Thirty-eight (38.4%) were female, 77.8% had hypertension, 21.2% had diabetes, 34.3% had dyslipidaemia, 19.8% were current smokers and 28.1% were current drinkers (Table 2). The baseline sTLR4 concentrations for the first, second, and third tertiles were 0.77 ± 0.31 ng/mL, 1.82 ± 0.44 ng/mL, and 4.62 ± 2.21 ng/mL, respectively. No clinical, biochemical or radiological differences were observed among ICH patients when stratified by sTLR tertiles, and no significant associations were found between the studied variables and baseline sTLR4 levels in the simple linear regression.

**Table 2:** Distribution of clinical characteristics across different sTLR4 levels and simple linear regression analyses for sTLR4

		sTLR4 tercile (ng/mL)				Simple linear regression		
	Overall (*n* = 99)	Lower (0.77 ± 0.31) (*n* = 33)	Middle (1.82 ± 0.44) (*n* = 33)	Higher (4.62 ± 2.21) (*n* = 33)	*p*-value	β	95% CI	*p*-value
Age, years	69.7 ± 12.9	67.7 ± 13.4	70.3 ± 15.0	71.2 ± 10.1	0.532	0.001	-0.031–0.034	0.932
Female sex	38 (38.4)	10 (30.3)	16 (48.5)	12 (36.4)	0.352	-0.034	-0.895–0.827	0.937
Hypertension	77 (77.8)	26 (78.8)	23 (69.7)	28 (84.8)	0.371	0.400	-0.604–1.404	0.431
Diabetes mellitus	21 (21.2)	9 (27.3)	4 (12.1)	8 (24.2)	0.298	-0.098	-1.122–0.926	0.849
Dyslipidaemia	34 (34.3)	11 (33.3)	10 (30.3)	13 (39.4)	0.805	-0.071	-0.953–0.810	0.873
Current smoking	19 (19.8)	9 (29)	5 (15.2)	5 (15.6)	0.293	-0.190	-1.265–0.885	0.726
Current drinking	27 (28.1)	9 (33.3)	10 (30.3)	8 (29.6)	0.921	0.041	-0.912–0.995	0.932
BP at admission								
Systolic, mmHg	169.0 [150.0–189.5]	162.0 [150.0–184.0]	171.0 [157.0–192.0]	170.0 [143.0–185.5]	0.656	0.003	-0.013–0.018	0.797
Diastolic, mmHg	87.0 [73.0–103.0]	90.5 [68.3–105.8]	90.0 [75.0 -100.0]	86.5 [74.8–103.0]	0.945	0.004	-0.019–0.026	0.700
Antiplatelet treatment	22 (22.2)	7 (21.2)	5 (15.2)	10 (30.3)	0.371	-0.158	-1.165–0.849	0.756
Anticoagulant treatment	15 (15.2)	2 (13.3)	6 (18.2)	7 (21.2)	0.205	0.730	-0.429–1.889	0.214
Leukocyte count, 10^9^/L	8.7 ± 2.8	8.8 ± 2.8	8.4 ± 2.6	8.8 ± 3.0	0.728	0.014	-0.137–0.166	0.853
Fibrinogen, ng/dL	416.3 ± 103.1	420.9 ± 104.9	425.2 ± 111.7	402.9 ± 93.6	0.653	-0.002	-0.007–0.002	0.224
SII	1211.1 ± 1290.1	1033.7 ± 1074.5	1173.3 ± 1071.4	1420.9 ± 1644.6	0.476	0.000	0.000–0.001	0.121
NIHSS at admission	9.0 [5.0–19.0]	8.0 [4.0–18.0]	11.0 [6.0–18.5]	8.0 [5.0–19.0]	0.572	0.003	-0.056–0.062	0.910
Onset time to CT, hours	3.4 ± 2.7	3.69 ± 2.98	2.97 ± 2.33	3.57 ± 2.66	0.497	0.089	-0.068–0.246	0.263
ICH volume, mL	8.9 [3.2–19.6]	10.8 [3.3–21.5]	8.8 [3.6–18.4]	7.1 [2.4–25.9]	0.993	0.012	-0.012–0.036	0.332
Intraventricular haemorrhage	22 (22.2)	7 (21.2)	5 (15.2)	10 (30.3)	0.371	0.851	-0.141–1.844	0.092
Lobar ICH location	21 (21.2)	8 (24.2)	6 (18.2)	7 (21.2)	0.952	0.287	-0.736–1.309	0.579
Spot sign	19 (19.2)	5 (15.2)	7 (21.2)	7 (21.2)	0.853	0.431	-0.629–1.490	0.422
Early neurologic deterioration	13 (13.1)	5 (15.2)	3 (9.1)	5 (15.2)	0.807	-0.247	-1.486–0.992	0.693

Categorical variables are expressed as n (%) and numerical variables as mean ± standard deviation (normally distributed) or median [interquartile range] (non-normally distributed). BP, blood pressure; SII, Systemic Immune-Inflammation Index; CT, computed tomography; ICH, intracerebral haemorrhage; NIHSS, National Institutes of Health Stroke Scale. P-value obtained by the Chi-square test, Fisher’s exact test, ANOVA, or the Kruskal-Wallis test, as appropriate

### Temporal Profile of sTLR4 in ICH Patients With and Without HE

Among the 99 patients with ICH, 27 (27.3%) showed HE. Significant differences in NIHSS at admission, baseline ICH volume and presence of spot sign were observed between patients with and without HE. Patients with HE had higher NIHSS scores at admission (19.0 [7.0–21.0] vs. 8.0 [5.0–16.0], *p* = 0.004), larger baseline ICH volume (27.0 [13.3–43.8] vs. 5.9 [2.3–13.2], *p* < 0.001) and were more likely to present spot sign (37.0% vs. 12.5%, *p* < 0.010) (Table 3).

**Fig. 2:**
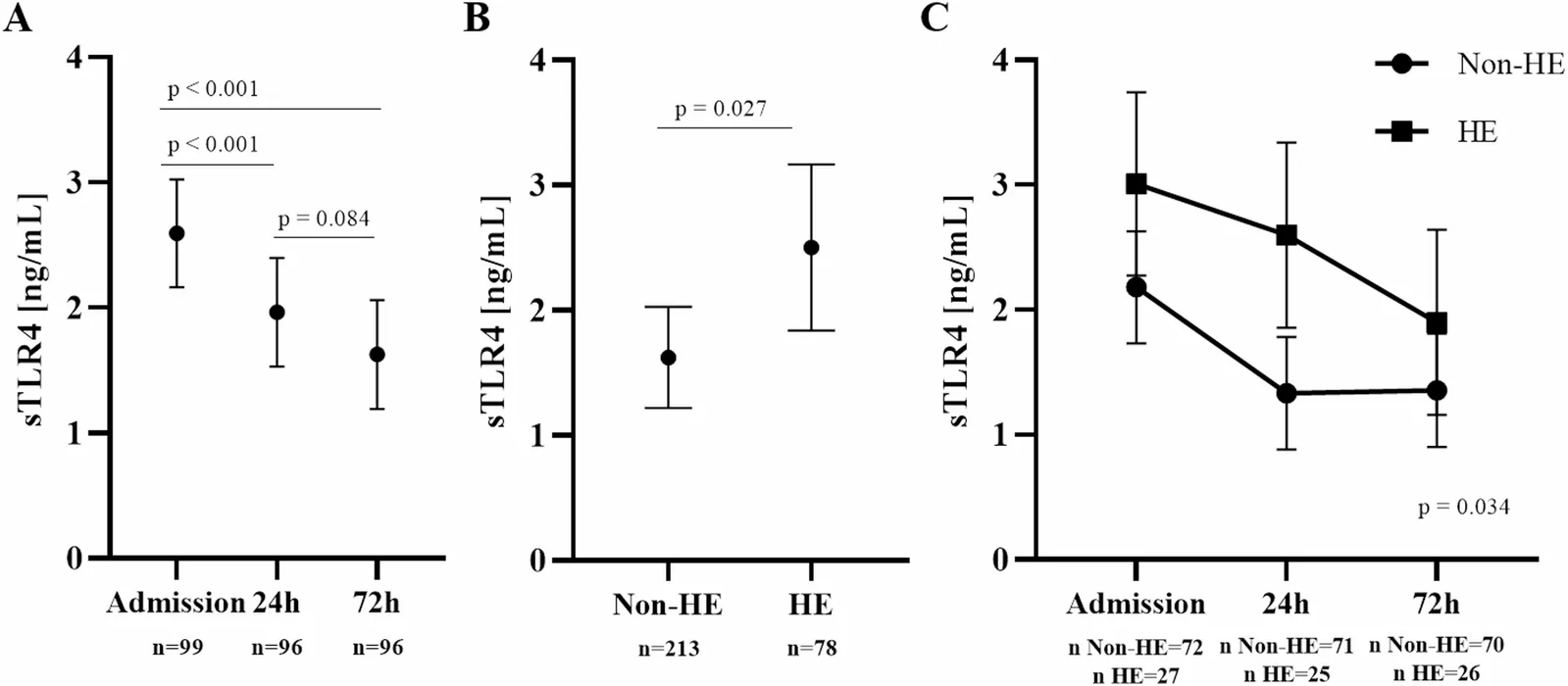
Linear mixed-effect model of sTLR4 levels in ICH patients: (**A**) over time (p-value obtained by t-test with bonferroni correction), (**B**) by haematoma expansion (HE) status (p-value obtained by t-test) and (**C**) temporal profile stratified by haematoma expansion status (p-value of the interaction obtained by Type III test of fixed effects (F-test)). Data are presented as mean (95% CI)

Linear mixed-effects models revealed significant effects of sampling time and HE status on sTLR4 levels, as well as a significant interaction between time and HE status (Table S1). Sampling time was significantly associated with sTLR4 levels (F(2,192.1) = 12.52, *p* < 0.001), with higher levels at admission followed by a progressive decline at 24 and 72 h (Fig. 2A). HE status was also significantly associated with sTLR4 concentrations (F(1, 106.2) = 5.03, *p* = 0.027), with higher overall levels observed in patients with HE compared to those without HE (Fig. 2B). Importantly, a significant time x HE interaction was observed (F(2, 192.1) = 3.45, *p* = 0.034), indicating different longitudinal trajectories according to HE status. Patients with HE showed a gradual decrease in sTLR4 levels over time, whereas patients without HE exhibited a sharper early decline followed by stabilisation (Fig. 2C).

**Table 3:** Baseline clinical characteristics, vascular risk factors and radiological parameters in ICH patients with and without HE

	Non-HE (*n* = 72)	HE (*n* = 27)	*p*-value
Age, years	68.4 ± 13.2	73.3 ± 11.8	0.094
Female sex	25 (34.7)	13 (48.1)	0.251
Hypertension	60 (83.3)	17 (63.0)	0.055
Diabetes mellitus	18 (25.0)	3 (11.1)	0.172
Dyslipidaemia	27 (37.5)	7 (25.9)	0.346
Current smoking	15 (21.7)	4 (14.8)	0.574
Current drinking	19 (27.5)	8 (29.6)	1.000
BP at admission			
Systolic, mmHg	171.0 [150.0–190.0]	165.0 [147.5–183.8]	0.466
Diastolic, mmHg	90.0 [71.5–104.5]	86.0 [78.8–97.3]	0.844
Antiplatelet treatment	16 (22.2)	6 (22.2)	1.000
Anticoagulant treatment	12 (16.7)	3 (11.1)	0.754
Leukocyte count, 10^9^/L	7.7 [6.7–9.8]	8.2 [6.4–10.3]	0.798
Fibrinogen, ng/dL	409.0 [344.3–492.3]	387.0 [344.0–435.0]	0.398
SII	724.5 [450.6–1402.5]	660.0 [456.0–1174.5]	0.978
NIHSS at admission	8.0 [5.0–16.0]	19.0 [7.0–21.0]	**0.004**
Onset time to CT, hours	2.3 [1.8–4.0]	2.0 [1.7–5.5]	0.680
ICH volume, mL	5.9 [2.3–13.2]	27.0 [13.3–43.8]	**< 0.001**
Intraventricular haemorrhage	13 (18.1)	9 (33.3)	0.113
Lobar ICH location	12 (16.7)	9 (33.3)	0.097
Spot sign	9 (12.5)	10 (37.0)	**0.010**
Early neurological deterioration	8 (11.1)	5 (18.5)	0.334

Categorical variables are expressed as n (%) and numerical variables as mean ± standard deviation (normally distributed) or median [interquartile range] (non-normally distributed). *BP *blood pressure, *SII *Systemic Immune-Inflammation Index, *CT* computed tomography, *HE* haematoma expansion, *ICH* intracerebral haemorrhage, *NIHSS* National Institutes of Health Stroke Scale. P-value obtained by the Chi-square test, Fisher’s exact test, Student’s t-test, or Mann-Whitney U test, as appropriate

### Association of sTLR4 Concentration at Admission and HE

In the univariable logistic regression analysis, absence of a previous history of hypertension (OR 0.340, 95% CI 0.125–0.922, *p* = 0.034), higher NIHSS score at admission (OR 1.108, 95% CI 1.036–1.185, *p* = 0.003), higher baseline ICH volume (OR 1.073, 95% CI 1.037–1.111, *p* < 0.001) and the presence of a spot sign (OR 4.118, 95% CI 1.444–11.741, *p* = 0.008) were significantly associated with HE. Older age (OR 1.032, 95% CI 0.994–1.071, *p* = 0.097), lobar ICH location (OR 2.500, 95% CI 0.909–6.879, *p* = 0.076), and higher sTLR4 levels at admission (OR 1.192, 95% CI 0.972–1.461, *p* = 0.092) showed a trend toward an association with HE (Table 4).

In the multivariable logistic regression analysis including age, NIHSS at admission, baseline ICH volume, spot sign, ICH location and sTLR4 levels at admission, only baseline ICH volume remained independently associated with HE in the final model. However, sTLR4 levels and baseline ICH volume were the two variables retained in the penultimate step of the selection process (Table 4 and Table S2). This finding justified further to evaluate the independent contribution of sTLR4 levels at admission, in combination with ICH volume, to the prognosis of HE.

**Table 4:** Univariable and multivariable logistic regression analysis for factors associated with HE

Variables	Univariable analysis			Multivariable analysis		
	OR	95% CI	*p*-value	OR	95% CI	*p*-value
Age, years	1.032	0.994–1.071	*0.097*	*-*		
Female sex	1.746	0.712–4.282	0.224			
Hypertension	0.340	0.125–0.922	**0.034**			
Diabetes mellitus	0.375	0.101–1.395	0.143			
Dyslipidaemia	0.583	0.218–1.561	0.283			
Current smoking	0.626	0.187–2.091	0.447			
Current drinking	1.108	0.146–2.954	0.838			
BP at admission						
Systolic, mmHg	0.996	0.979–1.012	0.591			
Diastolic, mmHg	1.001	0.978–1.024	0.949			
Antiplatelet treatment	1.000	0.345–2.898	1.000			
Anticoagulant treatment	0.625	0.162–2.413	0.495			
Leukocyte count, 10^9^/L	1.042	0.892–1.217	0.602			
Fibrinogen, ng/dL	0.998	0.993–1.002	0.372			
SII	1.000	1.000–1.001	0.272			
NIHSS at admission	1.108	1.036–1.185	**0.003**	-		
Onset time to CT, hours	1.033	0.878–1.215	0.695			
ICH volume, mL	1.073	1.037–1.111	**< 0.001**	1.073^a^	1.037 – 0.111	**< 0.001**
				1.073^b^	1.035 – 0.111	**< 0.001**
Intraventricular haemorrhage	2.269	0.834–6.171	0.108			
Lobar ICH location	2.500	0.909–6.879	*0.076*	-		
Spot sign	4.118	1.444–11.741	**0.008**	*-*		
Early neurologic deterioration	1.818	0.538–6.145	0.336			
sTLR4 at admission, ng/mL	1.192	0.972–1.461	*0.092*	1.164^b^	-0.928–1.460	0.189

^a^ Variables retained in the last step of the backward conditional model. ^b^ Variables retained in the penultimate step of the backward conditional model. *BP* blood pressure, *SII *Systemic Immune-Inflammation Index, *CI* confidence interval, *ICH *intracerebral haemorrhage, *NIHSS* National Institutes of Health Stroke Scale, *OR*, odds ratio, *sTLR4 *soluble Toll-like receptor 4

**Fig. 3:**
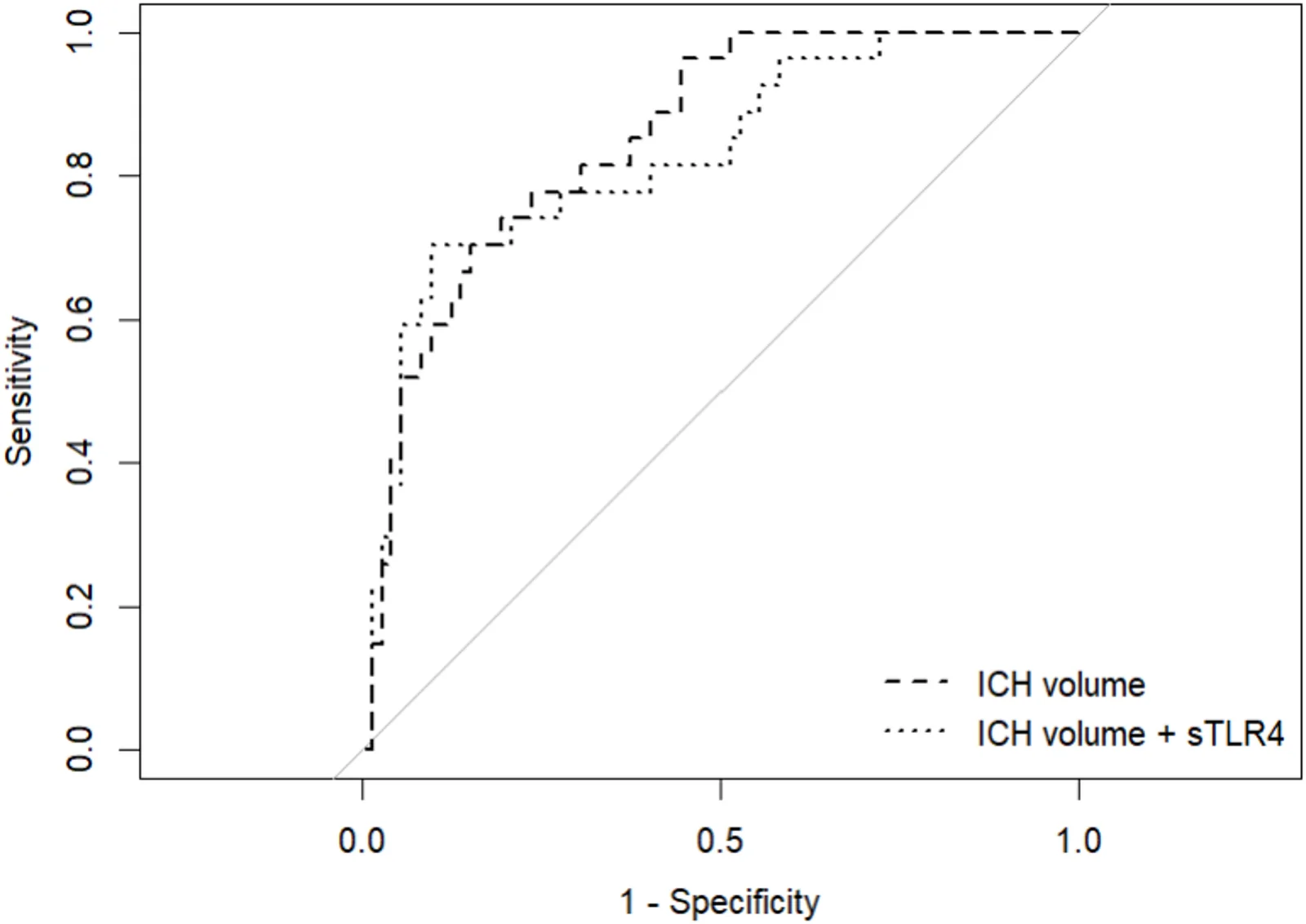
ROC curves comparing the AUCs of baseline ICH volume alone and in combination with serum sTLR4 levels at admission. The curve above the diagonal shows the true-positive against the false-negative rate for determination of possible cutoff values of a prediction algorithm as a trade-off between sensitivity and specificity

The ROC curve for the baseline ICH volume model yielded an AUC of 0.852 (95% CI 0.775–0.930), whereas the model combining baseline ICH volume and sTLR4 levels yielded an AUC of 0.829 (95% CI 0.734–0.923) (Table 5; Fig. 3). The optimal cutoff points for each model prognosticating HE were 17.13 mL for baseline ICH volume and a probability of 0.312 for the combined model. These cutoffs corresponded to a sensitivity of 70.4% for both models, a specificity of 84.7% and 90.3%, a PPV of 60.5% and 70.8%, and a NPV of 89.6% and 90.1%, respectively.

**Table 5:** Perfomance metrics of prognostic models for HE

	AUC	95% CI	Sensitivity (%)	Specificity (%)	PPV (%)	NPV (%)
Baseline ICH volume	0.852	0.775–0.930	70.4	84.7	60.5	89.6
Baseline ICH volume + sTLR4 levels	0.829	0.734–0.923	70.4	90.3	70.8	90.1

## Discussion

Understanding the underlying mechanisms of HE is crucial for the identification of prognostic biomarkers of this event and the development of novel therapeutic targets to mitigate its effects. To our knowledge, this is the first study aiming to characterise the temporal profile of serum sTLR4 across the hyperacute and acute phases of ICH in patients with and without HE, and to evaluate the potential of baseline serum sTLR4 levels as a prognostic biomarker of HE. Serum sTLR4 levels were significantly higher in patients with ICH than in non-stroke controls and decreased significantly from baseline to 24 and 72 h after ICH onset. Notably, patients with HE presented significantly higher levels of sTLR4 and a distinct temporal profile compared with patients without HE, maintaining elevated levels of sTLR4 for a longer time. Patients with HE showed a gradual decrease in sTLR4 levels from baseline to 72 h, while those without HE exhibited an early decline at 24 h followed by stabilisation. Moreover, although our findings indicate that baseline ICH volume remains the most robust independent prognostic factor for HE, the addition of baseline sTLR4 levels, despite slightly reducing the overall discriminative ability (AUC), improved classification performance at the clinically relevant decision threshold, increasing both specificity and PPV for HE risk stratification.

Higher levels of sTLR4 in ICH patients with respect to non-stroke controls are in agreement with a previous study in Asian population [24]. It has been hypothesised that its release may arise primarily from TLR4 overactivation as a form of negative-feedback, as sTLR4 acts as a decoy interacting with TLR4 ligands and thereby inhibiting TLR4 signalling [28]. Therefore, in ICH, higher levels of sTLR4 may reflect the activation of immune innate responses triggered by the haemorrhage. Indeed, in a mouse model it has been described that following the initial vessel rupture, endogenous ligands released from the blood activated TLR4 expression in microglia, triggering an inflammatory cascade [29]. Moreover, it has also been proposed that a smaller proportion of sTLR4 may originate from membrane shedding during necrotic cell death [30]. Thus, in ICH, a small amount of sTLR4 may be released from necrotic brain cells that reach peripheral circulation as a result of a compromised blood brain barrier (BBB).

Concerning the temporal profile of serum sTLR4 in ICH patients, the subsequent decline over 24–72 h might reflect resolution of the hyperacute phase and a gradual return toward baseline as the injury stabilises and inflammation diminishes. These temporal dynamics are consistent with a previous study in patients with ICH reporting a peripheral downregulation of TLR4 in neutrophils and monocytes from baseline to 7 days after stroke onset. In this study, the observation that patients with poor outcomes exhibited increased TLR4 expression at admission, followed by a progressive decline over time, supported the hypothesis that TLR4 play a pivotal role in triggering the exaggerated inflammatory response observed in the early phase of ICH. In line with this interpretation, previous studies conducted by our group have demonstrated that inflammatory markers measured during the early acute phase of ICH are associated with subsequent HE and unfavourable clinical outcomes [11–13]. Collectively, these data support the notion that the inflammatory response contributing to secondary brain injury may, at least in part, be a downstream consequence of TLR4 activation.

Our findings are consistent with a previous study that demonstrated that in perihaematomal tissue from patients who required haematoma evacuation surgery, TLR4 expression began to increase within the first 6 h, reaching its peak between 24 and 72 h after the ICH [31]. Therefore, circulating sTLR4 levels may reflect TLR4 activation in the brain, which could be implicated in the pathophysiology of HE. TLR4-mediated inflammation and perihaematomal oedema may increase the fragility of surrounding tissue, facilitating HE [6]. However, further studies are needed to confirm these results. Notably, TLR4 antagonist facilitated haematoma absorption by increasing the expression of CD36, which suggests that TLR4 may attenuate haematoma clearance in the acute phase of ICH [19]. Moreover, recent clinical data showed that higher sTLR4 levels were independently associated with poor functional outcomes at three months in ICH patients [24]. This association may be partly explained by the link between sTLR4 and HE, a key driver of poor prognosis. Importantly, although TLR4 overactivation is not specific to ICH and may be influenced by subclinical infections or other unrecognised systemic inflammatory conditions, we found no evidence that baseline sTLR4 levels were affected by the analysed potential confounders. Specifically, baseline clinical, biochemical and radiological characteristics, including fibrinogen levels, leukocyte count and the SII, did not differ significantly across sTLR4 tertiles and were not associated with baseline sTLR4 levels in simple linear regression analyses. These findings suggest that the distinct temporal profile of sTLR4 observed in patients who developed HE is unlikely to be driven by initial disease severity or other measured confounding factors. Together, these results strengthen the biological plausibility that sTLR4 reflects early TLR4-mediated inflammatory activity, which may contribute to mechanisms underlying HE.

The potential role of TLR4 in the pathophysiology of HE opens the possibility of applying therapies aimed at targeting this molecule in ICH patients at risk of developing HE. Indeed, a TLR4 antagonist (ApTOLL) has already been developed, and its safety and efficacy have been studied in a clinical trial including only ischaemic stroke patients, demonstrating a potentially meaningful clinical effect [32]. The results of the present study, considered in the context of existing evidence, suggest that treatment with ApTOLL warrants further investigation as a potential therapeutic approach in selected patients with ICH. While its role in preventing HE cannot be established from the current data, the observed associations support the hypothesis that early modulation of TLR4–mediated inflammation may be relevant in this setting. Given the prior demonstration of safety in ischaemic stroke, the feasibility of pre-hospital administration before brain CT could be explored in future studies, with the aim of facilitating earlier intervention within the hyperacute phase of ICH. The competitive advantage of ApTOLL in ICH, compared with small-molecule inhibitors such as TAK-242, lies in its extracellular specificity. Considering that harmful ligands such as heme and fibrin are present in the interstitial space following haemorrhage, an aptamer that blocks the receptor at its extracellular domain may be more effective in preventing the initiation of downstream signalling than a drug that must penetrate the cell to exert its effects [33].

Regarding the evaluation of baseline serum sTLR4 levels as a prognostic biomarker of HE, our findings are consistent with previous studies reporting that baseline ICH volume is independently associated with HE [4, 34]. Although sTLR4 did not emerge as an independent prognostic variable, its inclusion in the model provided complementary value, increasing specificity and PPV, while preserving NPV. These findings suggest that sTLR4 levels at admission may enhance the clinical utility of baseline ICH volume for stratifying patients into low- and high-risk groups for HE at the decision threshold used. Consequently, sTLR4 levels may be valuable for early risk stratification, primarily identifying patients unlikely to benefit from intensive anti-HE interventions or inclusion in clinical trials (NPV 90.1%), while also, to a lesser extent, helping identify those more likely to benefit (PPV 70.8%).

To understand the place of sTLR4 in the clinical workflow, it must be compared with current gold-standard imaging markers used to predict HE. While radiological signs provide immediate spatial information, biochemical markers like sTLR4 may offer a more dynamic picture of the biological propensity for further bleeding. The SS on CTA remains the most validated radiological predictor of HE. Nevertheless, despite its high specificity that reaches the 90%, the sensitivity of the SS is relatively modest, often reported around 57% [8]. This means that a significant portion of patients who do not demonstrate a SS will still experience HE. Notably, the model including baseline ICH volume and sTLR4 levels represents an improvement over the current gold standard, increasing the sensitivity to 70.4%. Further research is needed to establish strategies that combine clinical data with imaging and inflammatory biomarkers, which may improve the prediction of HE.

Several limitations should be acknowledged. The single-centre design and modest sample size.

may limit the generalisability of our findings. Furthermore, the modest number of events resulted in limited statistical precision. Additionally, because sTLR4 is not specific to ICH, residual confounding from undiagnosed or comorbid conditions remains possible. Moreover, individuals with very large baseline haematoma volumes were more likely to die early, which prevented sample collection at subsequent time points. As a result, the full spectrum of disease severity may not be fully represented in our analysis, which introduces a survival bias. Consequently, the true association between sTLR4 levels and HE may be underestimated.

Overall, our findings provide new insights into the pathophysiology of HE, supporting a potential role of TLR4 in its development and highlighting it as a potential target for future therapeutic investigation. In addition, baseline sTLR4 levels may help in identifying ICH patients at higher and lower risk of developing HE, offering a complementary tool for early risk stratification.

## Supplementary Information

Below is the link to the electronic supplementary material.


Supplementary Material 1


## Data Availability

The datasets used and/or analysed during the current study are available from the corresponding author on reasonable request.

## Abbreviations

BBB: blood brain barrier
BP: blood pressure
CT: computed tomography
CTA: computed tomography angiography
ELISA: enzyme-linked immunosorbent assay
HE: haematoma expansion
ICH: intracerebral haemorrhage
mRS: modified Rankin Scale
NCCT: non-contrast computed tomography
NIHSS: National Institutes of Health Stroke Scale
SS: spot sign
sTLR4: soluble Toll-like receptor 4
TLR4: Toll-like receptor 4
