# Supplementary material for: Temporal Profile of Soluble TLR4 and its Association with Intracerebral Haemorrhage Expansion
Source: Transl Stroke Res. 2026 Jul 20;17(4):82. doi: 10.1007/s12975-026-01473-2 (PMC13385087; doi:10.1007/s12975-026-01473-2)
Supplement: Supplementary file 1 — Supplementary Material 1 [file 12975_2026_1473_MOESM1_ESM.docx]

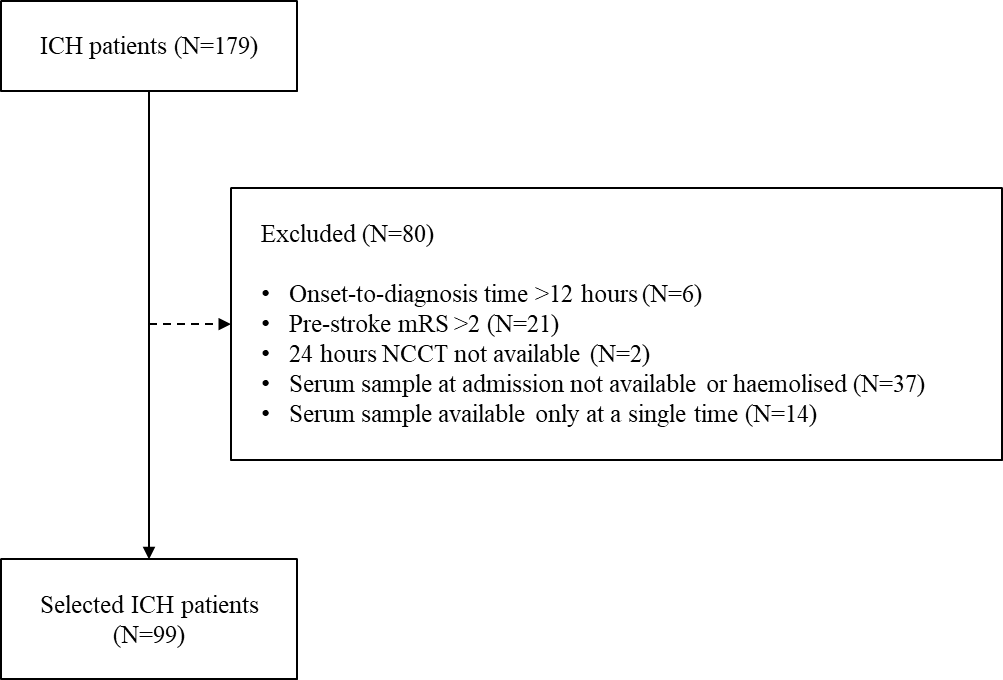


**Figure S1.** Flowchart of patient selection for the study.

**Table S1.** Type III tests of fixed effects examining the influence of sample time and HE on sTLR4 levels in the linear mixed model.

| Source | Numerator df | Denominator df | F value | p-value |
| --- | --- | --- | --- | --- |
| Intercept | 1 | 106.198 | 110.715 | < 0.001 |
| Sample time | 2 | 192.052 | 12.519 | < 0.001 |
| HE | 1 | 106.198 | 5.033 | 0.027 |
| Sample time x HE | 2 | 192.052 | 3.453 | 0.034 |

HE, haematoma expansion.

**Table S2. Multivariable backward conditional model**

| Step | Variables | OR | 95% CI | p-value |
| --- | --- | --- | --- | --- |
| 1 | Age, years | 1.024 | 0.981 – 1.068 | 0.279 |
|  | NIHSS at admission | 1.033 | 0.937 – 1.139 | 0.509 |
|  | ICH volume, mL | 1.056 | 1.010 – 1.105 | **0.017** |
|  | Spot sign | 1.860 | 0.492 – 7.035 | 0.361 |
|  | Lobar ICH location | 1.388 | 0.321 – 5.998 | 0.660 |
|  | sTLR4 at admission, ng/mL | 1.165 | 0.926 – 1.467 | 0.192 |
| 2 | Age, years | 1.025 | 0.982 – 1.069 | 0.258 |
|  | NIHSS at admission | 1.023 | 0.938 – 1.116 | 0.605 |
|  | ICH volume, mL | 1.061 | 1.019 – 1.104 | **0.004** |
|  | Spot sign | 1.800 | 0.483 – 6.717 | 0.381 |
|  | sTLR4 at admission, ng/mL | 1.165 | 0.926 – 1.465 | 0.192 |
| 3 | Age, years | 1.025 | 0.983 – 1.069 | 0.247 |
|  | ICH volume, mL | 1.066 | 1.028 – 1.106 | **0.001** |
|  | Spot sign | 1.870 | 0.506 – 6.907 | 0.348 |
|  | sTLR4 at admission, ng/mL | 1.159 | 0.923 – 1.457 | 0.205 |
| 4 | Age, years | 1.028 | 0.986 – 1.072 | 0.192 |
|  | ICH volume, mL | 1.071 | 1.033 – 1.110 | **< 0.001** |
|  | sTLR4 at admission, ng/mL | 1.174 | 0.937 – 1.471 | 0.164 |
| 5 | ICH volume, mL | 1.073 | 1.035 – 1.111 | **< 0.001** |
|  | sTLR4 at admission, ng/mL | 1.164 | 0.928 – 1.460 | 0.189 |
| 6 | ICH volume, mL | 1.073 | 1.037 – 1.111 | **< 0.001** |

CI, confidence interval; ICH, intracerebral haemorrhage; NIHSS, National Institutes of Health Stroke Scale; OR, odds ratio; sTLR4, soluble Toll-like receptor 4.
